# Autistic adults’ views on the design and processes within randomised controlled trials: The APRiCoT study

**DOI:** 10.1177/13623613231202432

**Published:** 2023-10-26

**Authors:** Lucy Beasant, Alba Realpe, Sarah Douglas, Lorcan Kenny, Dheeraj Rai, Nicola Mills

**Affiliations:** 1 University of Bristol, UK; 2 Bristol NIHR Biomedical Research Centre, UK; 3 Autistic Researcher, UK; 4 Autistica, UK; 5 Avon and Wiltshire Mental Health Partnership NHS Trust, UK

**Keywords:** autistic adults, co-production, learning disabilities, qualitative research, trial methodology

## Abstract

The purpose of this study is to explore the views of autistic adults on randomised controlled trials, specifically on processes such as randomisation and blinding, to understand the barriers and facilitators for recruiting autistic people to randomised controlled trials involving medications. We conducted one-to-one interviews with 49 autistic adults. Interviews were audio-recorded and analysed thematically. The participants found randomised controlled trial processes acceptable and linked positive attitudes towards randomised controlled trial participation to autistic peoples’ heightened sense of fairness and preference for evidence-driven knowledge. However, randomised controlled trial designs may be incompatible with a (1) preference for a controlled predictable world, (2) perceived vulnerability at physical and mental health levels and (3) history of misunderstanding and exclusion, crucially from healthcare professionals. Suggestions that emerged from our findings include efforts to co-produce research to nurture trust and adapting communication practices to improve access to trials. Autistic people are a highly motivated group to work with research teams to mitigate barriers to randomised controlled trial participation. We explored what psychosocial determinants play a role in the acceptability of randomised controlled trials to test interventions to improve quality of life and mental health in autistic adults. The study provides useful information that may help the design and conduct of more accessible trials with and for the autistic community.

**Lay abstract:**

Large randomised controlled trials are used to test healthcare treatments. Yet there are no large randomised controlled trials on effective treatments for common mental health issues affecting autistic adults. The purpose of this study was to learn what autistic adults think about randomised controlled trials in preparation for a randomised controlled trial testing a medication for anxiety. This means we wanted to know their opinions about the way randomised controlled trials are done, such as how people are chosen to be in the study and how the study is carried out. We did this by talking to 49 autistic adults individually and asking them questions. We found that most of the people we talked to were okay with the way randomised controlled trials are done. They thought it was fair and they liked that it was based on evidence. However, some autistic people might find it hard to take part in randomised controlled trials. Some people did not like the uncertainty of not knowing what treatment they would receive in a randomised controlled trial. Others felt too vulnerable and may have had bad experiences with healthcare in the past. We found that it is important to involve autistic people early on and at every stage when designing a clinical trial. Care about how clear and precise the study communication is will build trust and improve access to research. Our study indicates that it is possible to conduct large randomised controlled trials with and for autistic people. This can ultimately contribute to the improvement of healthcare outcomes for this population.

Autism is a common neurodevelopmental condition with an estimated prevalence between 1% and 2% of the child and adult populations worldwide (Maenner et al., 2023; World Health Organization, 2023; Zeidan et al., 2022). In the past two decades, autistic individuals who do not have an intellectual disability are increasingly being identified and diagnosed with autism (Lai & Baron-Cohen, 2015). This group has been referred to as a ‘lost generation’ due to the lack of services to identify and support them. Several recent studies have highlighted higher physical and mental health inequities, as well as a greater incidence of premature mortality in autistic adults compared with the general population (Croen et al., 2015; Hirvikoski et al., 2016; Rai et al., 2018; Schendel et al., 2016; Selten et al., 2015).

In 2019, improving healthcare for autistic people was identified as a clinical priority for the England’s National Health Service (NHS) over a 10-year period (NHS England, 2019). However, effective care will require a strong evidence base and despite the need, there has been very little research on effective support for the physical and mental health of autistic people. There is an increasing acknowledgement that evidence from intervention studies of the non-autistic population may not always be directly transferable to the autistic population. For example, commonly used psychological therapies without adaptation may not be as effective or may have different side-effect profiles with the autistic population in comparison to non-autistic people due to communication and neurocognitive differences (Linden et al., 2023; National Institute for Health and Care Excellence, 2012). Furthermore, autistic people may not have the same response to mental health medications (e.g. antidepressants) due to different neurotransmitter profiles (Muller et al., 2016). Well-conducted and adequately powered randomised controlled trials (RCTs) are the gold standard research design to understand the effectiveness of healthcare interventions. This type of design permits to investigate cause and effect between treatment and outcome, because it minimises bias by randomising participants and creating comparable groups (Hariton & Locascio, 2018). However, large RCTs on effective treatments for common mental health issues have not been carried out within the adult autism population (Brugha et al., 2015).

Many existing RCTs that have recruited autistic adults are based on a handful of participants (Green & Garg, 2018). There has been a perception that large-scale RCTs may be challenging to carry out with the autistic population. One potential issue regarding the feasibility of large RCTs is the acceptability of randomisation in this population. Intolerance of uncertainty is often a common experience for autistic people (Jenkinson et al., 2020; Kerns et al., 2014), and this aspect of their experience has been linked to clinically significant anxiety levels (Boulter et al., 2014). Uncertainty is one of the central concepts behind randomisation and blinding (i.e. the patient and/or researchers are not in control of, or in case of blinding, even aware of the treatment allocation). Therefore, it is plausible that autistic adults may find the uncertainty underpinning the process of randomisation and/or blinding unacceptable or difficult, potentially being a barrier to their participation in RCTs. However, we know of no published literature on how autistic people perceive these processes central to RCTs, and whether this may impact their participation in such studies. This information will be crucial to inform the design of future RCTs involving autistic people. The Autistic Adults and Randomised Controlled Trials (APRiCoT) study aimed to understand how autistic adults perceive randomisation and the components of RCTs, to improve understanding of the potential barriers and facilitators to recruitment to RCTs in this population. We particularly focused on aspects of RCTs involving medications, as this study was conducted in preparation for an RCT of Sertraline versus placebo for anxiety in autistic adults (Rai et al., 2023).

## Method

We used an in-depth one-to-one semi-structured interview design. Our perspective was informed by critical realism that proposes that all knowledge is situated within the historical, social and cultural conditions of production and dependent on social actors, including the investigators (Gorski, 2013). In consequence, qualitative research being a systematic study of speech and behaviour, is a valid method to explore how people act in a socially coordinated and interdependent world (Bandura, 2001). Furthermore, we subscribe to community-based participatory research, already in use within the autism community (Nicolaidis et al., 2011), which advocates for power sharing between researchers and communities researched, and recognises the validity of experiential knowledge (Tremblay et al., 2018).

### Community involvement statement

Our study team included an autistic adult (S.D.), a psychiatrist with clinical and research expertise involving autistic people (D.R.), experienced qualitative researchers (N.M., L.B., and A.R.) and the then Head of Research from an autism charity (L.K.), who all contributed to the study design, conduct, materials, interpretation and write-up. Ethical approval was obtained from the University of Bristol, Faculty of Health Sciences Ethics Committee (Ref. 93282).

### Participants

We interviewed 49 individuals between December 2019 and July 2020. Participants were 18 years or older, had a diagnosis of autism, understood the participant information sheet and consented to participate in a one-to-one interview with a researcher in English. Interviews lasted on average 54 min (ranging 23–105 min). We interviewed participants via telephone (*n* = 22), an online video conferencing platform (*n* = 17, e.g. Skype, Bluejeans, Zoom), e-mail (*n* = 8), face-to-face (*n* = 1) and SMS (*n* = 1), depending on their preference.

### Materials

We developed a semi-structured topic guide to ensure interviews covered the same issues but with sufficient flexibility to enable participants to raise areas they considered as important (Supplemental material 1). In designing the co-produced topic guide, we followed recommendations about how to adapt interviews to autistic people using scaffolding and visual–verbal prompting (Kenny et al., 2016; Norris et al., 2020). First, we asked interviewees to elaborate on the experience of being autistic (e.g. *we would first like to ask some questions about how, or if, being autistic affects your life*) and their previous experiences of research participation. Second, we referred to a document sent in advance of the interview and drafted by experienced methodologists from our study team (D.R., N.M., L.B. and A.R.), who have worked on optimising recruitment and informed consent to clinical trials. The easy-to-read summary document summarised the main concepts employed in RCTs, specifically randomisation, placebo and blinding (e.g. have you had a chance to read the document we sent you called ‘Explaining randomised controlled trials (RCTs)’ (please see Supplemental material 2). Finally, we discussed an example of a double-blinded placebo-controlled RCT for the treatment of anxiety to explore their motivations and barriers to participation (e.g. ‘I’d like to now ask about your views on participating in a specific RCT and then I will ask you some questions about it’; Supplemental material 1). While conducting the interviews, the COVID-19 pandemic broke out, and we submitted an ethical amendment to add questions about the effects of the lockdown and possible impact on future RCT participation; these findings are reported elsewhere (Realpe et al., 2023).

### Procedure

We invited volunteers to express interest in the APRiCoT study via social media and mailshot to our charity partner network subscribers, which links researchers to individuals interested in participating in autism research. Volunteers expressed an interest in participating by completing an online screening questionnaire which asked them to provide autism diagnosis as recorded in their autism assessment, the year they received this diagnosis, year of birth, gender, first section of their address, highest educational qualification, current employment status, preferred mode of contact and contact details. Those who met the inclusion criteria; adults (aged 18+ years) with a diagnosis of autism, who were willing to participate in a one-to-one interview in English, were eligible to participate. We sampled volunteers purposively to ensure maximum variation in relation to their gender, age, level of education and current employment status. Qualitative researchers (A.R. and L.B.) approached potential participants, answered any questions, sought to obtain informed consent and scheduled interviews according to participant’s communication preferences. Four members of the team (L.B., A.R., N.M. and D.R.) conducted the interviews, which were transcribed verbatim and de-identified. Interviewees received a gift voucher for their participation.

### Data analysis

Our approach followed the steps suggested in reflexive thematic analysis (Braun & Clarke, 2006, 2019). After reading interview transcripts repeatedly, two authors (L.B. and A.R.) developed initial codes from an inductive perspective assisted by specialised software NVIVO (QSR International Pty Ltd., 2018). They met regularly to build themes from the initial coding, refining them and discussing disagreements; they presented ongoing analysis at regular meetings with the wider research team, in particular to engage on reflexivity (Braun & Clarke, 2019). We reflected on how our different professional (research methods, clinical, advocacy) and personal (e.g. neurotypical vs neurodivergent) backgrounds influenced the interaction with participants. For example, three interviewers (N.M., A.R. and L.B.) had limited or no experience of interviewing autistic people, which led to confronting naïve expectations, learning about autism and making adjustments to interview styles. Sampling, data collection and analysis occurred cyclically until we considered we had enough information power to support our proposed categories and themes structure (Braun & Clarke, 2021; Malterud et al., 2016), which in our view reflected a nuanced and rich description that answered our research question. To help ensure data trustworthiness, a set of transcripts (10%) were doubled-coded by different members of the team (L.B., A.R. and N.M.).

## Results

### Sample

A total of 141 individuals expressed an interest in the study by completing the initial online screening questionnaire; 15 people did not meet the inclusion criteria, 32 were contacted to take part in an interview but either did not respond to contact (*n* = 27) or declined to take part (*n* = 5), and 45 were thanked for completing the questionnaire but were not invited to interview after we reached the end of the study. Those who were not invited to interview were similar to the interview sample in terms of age, level of education and current employment status. Only one-third of those who expressed an interest in the study were male, therefore a higher number of females who expressed an interest in the study were not invited to interview. We interviewed 49 autistic adults, aged 21–67 years. All but one participant was based in the United Kingdom. Half were employed (49%) and over two-thirds had college/bachelor’s degree-level education (69%; see Table 1). A complete set of demographic data is available in Supplemental material 3.

**Table 1.** Summary of demographics of interview participants.

Characteristic	*N*	(%)
Age	45*	(21–67)*
Gender		
Female	22	(45)
Male	24	(49)
Non-binary	3	(6)
Employment status		
Full time/self-employed	20	(41)
Part time	6	(12)
Unemployed/disabled	14	(29)
Student/volunteer	4	(8)
Retired	5	(10)
Highest educational qualification		
Pre-degree	14	(29)
College/bachelor degree	15	(31)
Postgraduate	20	(41)
Self-reported diagnosis		
Autism spectrum condition	23	(47)
Asperger’s syndrome	26	(53)

*Note: N* = number of participants; % = percentage of participants.

* Average age/Age range.

### Findings

#### Context of reported views on RCT participation

We reflected with our study participants about how autistic traits may influence their willingness to take part in RCTs. They highlighted that for autistic people, like themselves, who do not have moderate to severe learning disabilities (LD), concerns about research participation may not differ greatly from those of neurotypical populations. Most interviewees indicated that participation in an RCT would generally ‘greatly depend on how the RCT is designed, who is running it, how it is funded, and what it is trying to do’ (P48).

Study participants told us that many autistic people without LD may not be research-naïve and would have a basic understanding of research principles and processes (e.g. randomisation, blinding, etc.), either because of their professional interests or extensive reading. They explained that often autistic people have the capacity to pursue their interests in greater depths than neurotypical individuals; as a result, some neurodivergent people were naturally inclined to professions that required attention to detail and data processing, including scientific research. In our study, participants were a highly educated sample – 35 of the 49 had a first degree or higher with a small proportion having a degree in science and working at a university. Most interviewees were familiar with research principles and terminology, they could appreciate the value of RCTs to ‘measure the effectiveness of potential treatment’ (P26) and recognised the advantage of RCTs to obtain unbiased high-quality evidence. In relation to risk, most participants were reassured by the knowledge that trials undergo strict ethics processes:

I would like to know that the risks have been assessed but I guess they would be, otherwise ethical approval wouldn’t have been granted because I know what is entailed. It’s not an easy hoop to go through, is it? For me, that would be fine. (P3)

According to our participants, challenges to participation were likely to be related to psychosocial barriers associated with their day-to-day experience, which have been present for most of their lives. Chiefly, they mentioned ‘living with uncertainty’ (P31), especially in relation to social interaction and communication with others as well as being susceptible to experience intense sensory issues. Nevertheless, they insisted on the importance of autistic people being enrolled, and participating in all aspects of research.

**Figure 1.**
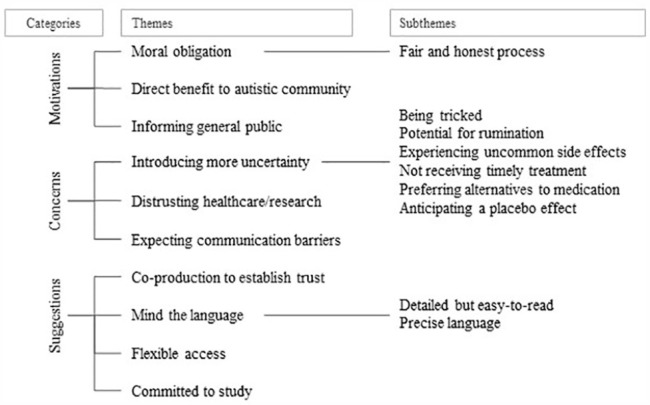
Themes and sub-themes from study interviews (*N* = 49).

Participants shared a wealth of diverse views and experiences about research participation, yet we found some commonalities that were organised in a structure of categories, themes and sub-themes illustrated in Figure 1 and described below.

#### Autistic people were highly motivated to participate in research

Interviewees talked about altruistic reasons to take part in research. We grouped their reasons into three themes noting slight variations between participants. They would consider research participation because of (1) moral obligation, (2) direct benefit to the autistic community and (3) its potential to inform and change society.

##### Moral obligation

Interviewees conveyed a strong sense of motivation to participate driven by ‘duty’ and ‘striving to do the right thing’:

It was my duty to do it [participate in research], regardless of the topic. I feel obliged with a medical condition to try and improve research and help with research. (P33)

They recognised that being autistic, and the accompanying sense of duty and desire for fairness, may facilitate the participation decision. Consistently, about two-thirds of interviewees had participated in studies in the past:

Finding out information is quite an appealing sort of thing and altruism I think is part of the condition as well. And I think knowing that you would have the chance to do something that might help yourself, and also help everybody else, is a very appealing prospect. (P44)

##### Fair and honest process

Several interviewees found randomisation and blinding acceptable processes because they maintained fairness and honesty in terms of allocating treatment and achieving a valid study outcome, characteristics they valued highly. Furthermore, a few study participants emphasised how a double-blinded trial, where participants as well as researchers do not know what treatment participants have been allocated to ensure equality between the parties, dissolving power imbalances:

Randomisation is used to get a broad spectrum of people for a study so that **it’s completely honest** (. . .) I like the fact that the results will be true and can’t have been made up. **Being autistic I like everything to be right and fair** (. . .) I’d not mind not knowing because that’s the whole **idea of complete fairness**, so not knowing would mean that everyone taking part would be equal. (P11)

#### Direct benefit to the autistic community

Interviewees’ altruistic motives to participate referred specifically to progressing medical research for the benefit of other autistic adults. In general, our interviewees remarked on the staggering lack of evidence on effective treatments to relieve mental health issues in autistic people such as stress, anxiety and depression, which are common experiences for this community:

[I agreed to the interview study] Because any way I can help the research community try and better understand what this autism is and how to help those who have it, I just take any opportunity to do that. (P20)

I would be pleased to help with this [RCT] research – I think it is important that I help where I can with anything that helps us to understand autism. (P19)

For some interviewees, an interest in helping other autistic people also extended to motivation to take part for themselves, with a hope that study participation might also result in personal insights or reflection:

Anxiety is a massive battle for me and I’ve never successfully been medicated. It’s something that, kind of, interests me as well. (P26)

#### Informing the general public about autism

A small number of interviewees mentioned the potential of research to inform and foster understanding among the general population. For these interviewees, by participating in research, they could contribute to changing attitudes that have traditionally excluded neurodivergent people from taking part in research:

I think it’s really important that people take part in research to help further the knowledge of it [autism] to make the world a more accepting place for [our kids]. Taking part in these sorts of programmes and getting that information then out there because eventually it does filter down to [the general population]. (P27)

#### Autistic people have some concerns about taking part in RCTs

Despite appreciating RCTs and a desire to progress science for the autistic community, many interviewees held concerns about participating when considering their unique lived experience as mentioned above. We grouped these issues into three themes: (1) introducing more uncertainty derived from RCT designs, (2) distrusting healthcare professionals and medical research and (3) expecting communication barriers between themselves and research teams.

##### Introducing more uncertainty

Study participants felt that taking part in an RCT could introduce uncertainty in their everyday lives, which in turn could raise their anxiety. Most interviewees preferred to follow established daily routines that offered a sense of control in everyday life to minimise uncertainty. Participating in an RCT was perceived to not only require having to adjust to new routines, people and environments, it would also introduce key sources of uncertainty derived from the study design. Interviewees reflected on how they would react to randomisation, blinding, active medication and placebo in the context of an RCT of a medication for reducing anxiety (Rai et al., 2023), which was then in the set-up stage.

##### Being tricked into participation

Most interviewees expressed concerns about differences in capacity to provide informed consent among autistic people. We recorded a few strong opinions related to feeling tricked or deceived, that would dissuade someone from considering research participation in an RCT. Interviewees felt that autistic people may feel especially vulnerable because some find it difficult to tell when ‘people are being false or being true’ (P32), or because they may feel their right ‘to be able to make the decision for yourself without influence from parents, carers or others in your life’(P3) has been negated in the past. Interviewees highlighted that researchers have the responsibility of communicating effectively, providing accessible material and enabling autistic people ‘to feel motivated and [find out] how to get involved without using undue influence to participate like an army-enlistment-type campaign directed at autistic people, I think it is a very dangerous ethical grey area’ (P37).

##### Potential for rumination

A few interviewees thought if they were blinded to the treatment, they may spend excessive time thinking whether their reactions were due to the active medication, placebo or a more general deterioration in their condition – ‘The uncertainty of whether I was taking the placebo or taking the regular one would drive me crazy and wake me up at night’ (P38), exacerbating their reservations about medication trials.

##### Experiencing uncommon side effects

The potential of experiencing side effects concerned interviewees, who cited effects from their own experience such as excessive weight gain, changes in perception, fatigue and brain fog, as well as difficult withdrawal. Based on anecdotal accounts, many interviewees sustained the view that autistic individuals might react differently than neurotypical people to medication. They also observed that autistic people were more likely to be taking other medication for co-occurring conditions, which increases the chances of harmful medication interactions. This complex set of circumstances made participants feel more vulnerable and cautious about deciding to take part in an RCT:

I’ve read a lot of first-person accounts of people having different reactions to drugs and being sensitive to different kinds of foodstuffs. While that indicates that there is a need for additional research in this area, it’s not something . . . I feel very vulnerable about that. (P5)

A few people raised other specific medication concerns, for example, wanting to know if the medications contained animal derivatives.

##### Not receiving timely treatment

Despite understanding the reason for a placebo in an RCT, some participants felt they would be anxious or disappointed about the possibility that they might receive placebo instead of the active medication – ‘I think it would be a big waste of time. . . I would want the treatment group’ (P2). This stemmed from narratives of intense psychological suffering and a belief that an active intervention would be beneficial. This was expressed more so by those who had positive experiences of taking medication:

My chief concern as a participant would be that, if I were to enter a particular trial because I required its treatment or service, being randomised to the control group would mean I didn’t receive it. That problem would be exacerbated if I went to any expense to participate in the trial, or if it caused me any physical or psychological difficulty. I would worry about this if participating in an RCT. (P12)

##### Preferring alternatives to medication

A few interviewees considered the possibility of an active intervention being no better than placebo. The view that research into psychological interventions to support autistic adults’ mental health needs might be more preferable than medication interventions was also raised by some participants:

Giving someone medication is the much cheaper and more straightforward pattern, as opposed to giving someone more complex and more direct forms of intervention through psychology or specialist therapies. (P3)

Yet most accepted that those who are invited to participate in an RCT, have to ‘be relatively comfortable with the intervention it offered (. . .) and comfortable with not knowing whether or not I receive any particular one of the intervention (or control) options’ (P12).

##### Anticipating a placebo effect

Feelings about discovering they had been taking placebo at the end of the study varied from anticipating disconcert and confusion to happiness and surprise:

I can’t possibly imagine ever wanting to put myself in a situation where I might be thinking that I’m going to have side-effects, but I might be imagining them. (P5)

A few people recognised the potential for placebo to offer a positive learning experience, by providing an effective way to overcome anxiety. Other study participants were sceptical about autistic people experiencing a placebo effect due to their cognitive preference for facts – ‘everything is fact based (. . .) we don’t necessarily expect it to work’ (P7), while others thought there was a great capacity of the autistic brain to induce healing – ‘I do believe that the mind is powerful’ (P11).

#### Distrusting healthcare professionals and medical research

The majority of participants described negative experiences of past healthcare interactions which left them feeling that health professionals were not responsive to their needs and experiences as autistic adults. For example, some felt there was little recognition and appreciation that autistic people might react differently or be more sensitive to commonly prescribed medications due to their autism:

If [patients on the autism spectrum] are saying they’re having side effects, or saying they’re having problems, they’re not being difficult patients. (P4)

Some interviewees suggested that common experiences of historic and ongoing health inequities among autistic adults, and a lack of accessible healthcare had eroded trust in relation to medical professionals, and this had potential to translate into a lack of trust in health research in healthcare settings:

I can’t imagine deliberately putting myself in a situation where I have to interact with healthcare professionals, putting myself in that kind of physical environment, to have to take medication, to talk to people who have not known how to deal with mental health, basically, I’ve had some really poor experiences with health professionals over the course of my lifetime. (P5)

More importantly, a few study participants expressed concerns about autism research that traditionally proposed answering research questions from an ableist perspective. Within this context, the thought of RCTs provoked feelings ranging from uneasiness to righteous anger because of the historical context of ‘treating’ or ‘curing’ autism, and other injustices. Most interviewees were politically engaged and advocated for a fair share in society for autistic people like them. This could work in favour of or against a research study, depending on its assumptions and how well it is communicated with eligible participants. Our interviewees provided guidance that we have presented in the overcoming barriers to RCT participation section below.

#### Expecting communication barriers

Our interviewees worried about having to communicate with neurotypical individuals in a research context. These concerns, if not addressed, could deter autistic people from entering a study, or make their research experience an unpleasant one, diminishing the chances of them participating in further research. For example, interviewees had found filling out questionnaires ‘extremely irritating’ (P18) because they had not been adapted for autistic people. Other interviewees feared their views may not be respected or understood despite their wishes to be accurate because of elements in the experience out of their control:

Healthcare is the least accommodating of almost any service I’ve ever been to. Buzzing fluorescent lights, an absurdly loud ticking clock in every office (just that sound nearly caused a shutdown for me), and a 15-minute window to explain ‘how you feel’ and what your symptoms are to a person you’ve rarely ever met! Nearly impossible with alexithymia on top of worrying that a doctor is going to misinterpret what you say as ‘not that bad’ because you don’t ‘sound upset’ or ‘look upset’. And it’s hard to decide if it’s ‘pain’ or ‘discomfort’ or whatever else you’d use to describe pain. (P8)

Finally, even in our interviews, we observed a few people who had difficulties explaining their own understanding of RCT designs, and despite reassurance, felt frustrated with the interview process because they could not demonstrate their own knowledge.

### Overcoming barriers to RCT participation

Having articulated their concerns with the idea of participating in an RCT, participants shared their thoughts on how these could be mitigated. They felt that developing and conducting research in close collaboration with the autistic community would help to establish trust and they offered various strategies that would help to minimise anxieties about uncertainty.

### Co-production to establish trust

Participants perceived added value in including autistic people in decision-making at all stages of the research process, from generating research questions to the dissemination of findings. Knowing that autistic people had been involved in the development stages of an RCT was important for some participants, particularly in terms of trusting the foundations of the research study and in making the research more inclusive:

It’s important to recognise that simply putting something through a randomised control does not remove the capacity for inequalities, and sometimes bad science as well. [Progress will be made when research includes] more autistic people, more women, more people of colour doing research, more queer people. Diversity, both the people who are being researched and the researchers. (P10)

#### Strategies to minimise uncertainty

##### Mind the language

Our interviewees discussed the ways in which the uncertainty involved in participating in an RCT might be minimised. At the top of their priorities was paying attention to the language used to explain the research, a key principle for accessibility to a research study. Interviewees felt study transparency as well as assurance that the research tools were appropriate and relevant for completion by autistic people would help promote trust in the research. For example, a participant noted how difficult it is to adequately express gender diversity in questionnaires:

I’d like to see a ‘which gender do you identify as? Women/Man/Non-binary/Other’ and ‘which sex were you born as? Female/Male/Intersex’ so that there’s more options especially in terms of healthcare for transgender people. It would be very helpful to know that information in a drug study like this (i.e., hormone effects and such). (P8)

Interviewees wanted transparent information about processes such as blinding from the beginning, assurance that intervention exposure was only temporary with rights to withdraw preserved, and that the study results would be relevant enough to have an impact in the whole community:

Knowing [what] ethical considerations are in place. What’s going to be done with the research – How’s it going to be used? How’s it going to make life better for people hopefully. (P42)

##### Detailed but easy-to-read information

Our participants explained that information about the study and treatments should include what RCT participation would involve practically, for example, where they would go and who they would see. Information needed to strike a balance between being sufficiently detailed but succinct and appealing to read – ‘make sure it’s not too in-depth and too wordy because I just wouldn’t read it’ (P26). To help alleviate anxieties related to inherent uncertainties of research processes, participants wanted information about defined lines of communication and clear protocols for adverse event reporting at the start of the study:

[Information] to almost be able to walk through an environment in my mind, to think about the different interactions that I might have and how I might respond to those. I’d have to plan that really carefully. (P5)

##### Precise language

Our study participants insisted researchers should strive to use clear and precise language, for example, a few people commented on an error in the description of ‘blinding’ emailed to participants prior to the interview (Supplemental material 2), which was in fact a description of ‘double-blinding’:

When I read your piece paper, at the end it said about blinding. And my immediate thought was, well, the actual line description was really more double blinding, rather than blinding. (P21)

##### Flexible access

Most interviewees emphasised that access to research should be flexible and consistent because ‘how easy it is to take part will be a facto’ (P36). They suggested taking advantage of online platforms to communicate, without discounting low-key in-person contact in a relaxing environment:

Doing a lot of things via email, that I think will help, limiting the face-to-face contact. Also, then, I think it’s more relaxed without time pressures. Certainly that’s something. . . I don’t respond too well under time pressure. (P1)

##### Committed to the study

Various interviewees described how despite anxiety produced by the trial design, if they were satisfied with the information received and reassurance from the trial team, they would put aside their anxieties and commit to the study. Although autistic people may take longer to decide whether to take part, they argued that once committed to the study, any ongoing anxieties would not interfere with their participation:

I would not like not being sure that I would see any benefit from the study, as would be the case if I were randomised to receive no treatment. However, I would not be worried about receiving the treatment without knowing – because I would already have made myself comfortable with the intervention options and I would trust the study team to follow me closely if there were any associated risks. (P12)

## Discussion

In this study, we explored autistic adults’ views on RCT participation. Our participants reported finding RCT processes acceptable and linked positive attitudes towards RCT participation to autistic peoples’ heightened sense of fairness, preference for evidence-driven knowledge, and their belief in science as common good. However, RCT designs may be incompatible with a (1) preference for a controlled predictable world, (2) perceived vulnerability at physical and mental health levels and (3) history of misunderstanding and exclusion, crucially from healthcare professionals. Suggestions that emerged from our finding include efforts to co-produce research with members of the autistic community to nurture trust, and adapting communication practices (e.g. precise and detailed information, contact flexibility) to improve access to trials. Autistic adults without LD appeared to be a highly motivated group that understand the need for these studies and were willing to work with research teams to mitigate barriers to RCT participation.

To our knowledge, this is the first study to explore what psychosocial determinants play a role in the acceptability of RCTs to test interventions to improve quality of life and mental health in autistic adults. Our findings matched facilitators and barriers reported in three large reviews of determinants of trial participation in general and patient populations (Houghton et al., 2020; Ross et al., 1999; Sheridan et al., 2020). Similar to our results, while altruism, personal benefit and trust in research or researchers have been cited as motivators for trial participation, concerns about risks, uncertainty, dislike of randomisations, lack of trust of researchers and concrete logistical aspects of taking part represent barriers to potential trial participants. Yet psychosocial determinants are sensitive to individual and context-specific factors, especially in relation to barriers to participation (Sheridan et al., 2020). Previous studies that have explored autistic adults’ views on research participation in longitudinal studies highlighted the inadequacy of research processes (e.g. use of validated instruments, attending study appointments), implemented without considering autistic people’s neurodiversity and their unique cognitive, behaviour and sensory needs (Haas et al., 2016). Despite high levels of motivation to take part in research, autistic people may feel extremely anxious, confused or angry when confronted with inappropriate research processes and instruments. This is likely to discourage participation and have a bearing on the validity, reliability and real-world meaning of autism research (Jones, 2022; Nicolaidis et al., 2020). When considering RCTs, our study participants revealed different levels of tolerance to uncertainty, change and reliance on routines. Most people worked on plans that helped them to modulate their emotional reactions and behaviours. Amid this complexity, just like they do with other lifestyle decisions, autistic people reported needing to carefully weigh up decisions about taking part in research. This was evidenced in the depth of their discourse when analysing RCT processes and their cautious, and at times, sceptical views of research. At the same time, their reflections demonstrated care and empathy for other members of the autistic community. They strived to maintain fairness and promote honest communication. These findings have been consistently reported in studies and projects aimed at promoting and facilitating engagement between researchers and members of this community (Gowen et al., 2019; Haas et al., 2016; Jones, 2022; Nicolaidis et al., 2019). Although we highlight the importance of co-production of research with autistic members, it would be important to consider potential risks such as tokenism or considering co-producers to be a ‘uniform voice’ for the entire community. Our intention is to highlight the need for trialists to recognise that current trial design and conduct need adaptations consistent with specific challenges derived from the neurodiversity of a target population such as in the case of autistic people.

Indeed, our results concur with current evidence about the pivotal role of study communication in participation in RCTs, which can sway people to take part or not (Houghton et al., 2020). Our findings suggest that in RCTs involving the autistic community the role of communication is critical and must be prioritised during the design and conduct of such studies. Difficulties with language and social communication are common experiences for autistic people (Lai & Baron-Cohen, 2015), and are often neglected when implementing research processes (Nicolaidis et al., 2020). Trial teams could focus on delivering information but also effectively facilitate communication of autistic participants with researchers, for example, by training recruiters on communication skills that would enable autistic people express themselves. With the potential for RCT designs to increase feelings of uncertainty, trialists need to ensure they have robust informed consent and safety protocols. They need to put in place accessible support systems at all points in the RCT process. All these actions will require patience and understanding but they are likely to be rewarded with enhanced motivation for continuous participation.

Finally, our study adds to previous research that used the technique of asking about hypothetical trial scenarios to the public, which has yielded important insights on how to improve informed consent to trials in the general population (Kerr, 2004). Yet, it is important to recognise that individuals’ decisions to participate in a prospective trial are likely to involve other context-specific factors (Wade et al., 2009).

### Limitations

Individuals who expressed an interest in this study were recruited via a charity that promotes autism research; most were well-educated, some had participated in research before (though not in RCTs) and some had a scientific background. These characteristics may have enhanced appreciation of the value of RCTs, and the processes involved. The study did not include people with intellectual disabilities, and we did not collect data on participants’ ethnicity or co-occurring physical or mental health conditions. Our recruitment strategy potentially excluded harder to reach members of the autistic community from lower socio-demographic groups. Therefore, our sample may have excluded people with less interest in research participation and those with greater support needs. Although we missed opportunities to add diversity to our sample, we achieved a balanced and diverse sample by targeting a wide range of characteristics from the survey data we collected (e.g. age group, gender, education level). Our sample of 49 in-depth interviews with autistic adults represents a large group when compared with many qualitative studies with the autistic community with typically smaller numbers.

### Conclusion

This study provides novel information about challenges and the need for adaptations to trial design and conduct derived from specific aspects of neurodiversity of a target population such as in the case of autistic people. We hope our work contributes to the successful co-production and conduct of RCTs with autistic people.

## Supplemental Material

sj-docx-1-aut-10.1177_13623613231202432 – Supplemental material for Autistic adults’ views on the design and processes within randomised controlled trials: The APRiCoT study Supplemental material, sj-docx-1-aut-10.1177_13623613231202432 for Autistic adults’ views on the design and processes within randomised controlled trials: The APRiCoT study by Lucy Beasant, Alba Realpe, Sarah Douglas, Lorcan Kenny, Dheeraj Rai and Nicola Mills in Autism
