# Supplementary material for: Autistic adults’ views on the design and processes within randomised controlled trials: The APRiCoT study
Source: Autism. 2023 Oct 26;28(6):1540–50. doi: 10.1177/13623613231202432 (PMC11134970; doi:10.1177/13623613231202432)
Supplement: sj-docx-1-aut-10.1177_13623613231202432 – Supplemental material for Autistic adults’ views on the design and processes within randomised controlled trials: The APRiCoT study [file sj-docx-1-aut-10.1177_13623613231202432.docx]

# Supplemental Materials

## Supplemental material 1

**Interview Topic guide**

## Part A. Introduction, consent and background

Thanks, introduce self, ASK HOW PARTICIPANT WOULD PREFER TO BE ADDRESSED, re-state purpose of the interview:

- **Aim:** to understand what adults with a diagnosis of autism think about participating in a randomised controlled trial (RCT).
- Mention recording of interview with permission, data confidentiality/ anonymisation and right to withdrawal at any point without giving a reason.
- Ask if any questions on the above.
- Record verbal consent to each question on consent form

**To start with we would like to briefly ask how, or if, being autistic affects your life** (e.g., do you have any routines you like to stick to in everyday life?

## Part B. Views on research studies

**I am now going to ask you about your views on research studies.**

- Are you the kind of person who would usually volunteer to take part in a research study if you were offered the opportunity? *[Probe: Why? Prior experience? Factors important to make decision (e.g. topic, team expertise, accommodate needs)?]*
- What could researchers do to enable autistic people participate in research that may help improve their well-being? *[Probe: thoughts on retaining changes even if not strictly required (e.g. online testing, remote consent)*

## Part C. Understanding RCTs

**I’d like to now ask about your understanding of an RCT and your views on it**

- Before you received information about this study, had you ever heard of a randomised controlled trial (RCT)? It may also be called a randomised clinical trial or study.
- We sent you a document called “Explaining randomised controlled trials (RCTs)” did you have time to read it?

#### General views on participating in RCTs:

- Does it make sense to you why RCTs are done? Understand the purpose of them?
- Does it make sense why they are done like this? i.e. why treatment is randomised, why an inactive treatment (placebo) may be used, and why the patient and doctor may not know what treatment is given (blinded)?

## Part D. Views on participating in a specific RCT

**I’d like to now ask about your views on participating in a specific RCT and then I will ask you some questions about it.**

| Researchers would like to find out if a treatment for anxiety (called sertraline) is an effective treatment for anxiety in autistic adults. They know that the treatment works in the non-autistic population but they do not know if it will treat anxiety in autistic people. They would like to do an RCT to find out.  In the RCT, autistic people who have problems with anxiety will be allocated to receive either Sertraline (the real/‘active’ medication) or a ‘Placebo’ (inactive medication). This process is done in a way that everyone has an equal chance of receiving either the active medication or placebo (randomisation).  Neither the person in the study or the doctor will know what medication has been allocated so that perceptions about the medication do not bias the study results (it’s a blinded RCT).  The person in the study will take the sertraline or the placebo for a year without knowing which one it is.  In this time they will have questionnaires to fill out and they will be closely monitored to check that they are doing ok. They can withdraw from the RCT at any time without giving a reason. |
| --- |

#### Views on participating in a specific RCT:

- What would you think if you were invited to take part in this RCT?
- What, if anything, might you like about this?
- What, if anything, might concern you about this?
- Do you think you would participate or not? Why?
- What do you think about not knowing what treatment you are taking in a blinded RCT like this?
- Would it make a difference if you didn’t know the treatment you were taking for a short time (perhaps a month) or a longer time (perhaps a year)?
- When the study finishes and you find out you were in the placebo group – what would you think about that?
- Have you ever taken medication for anxiety or depression (past/currently)? Experience?
- Do you know anyone who in past/currently takes sertraline or medications for anxiety or depression? [from autistic or neuro-typical population?] Their experience?
- Who/what do you trust in terms of getting information about new or existing medications? (GP, Internet etc)

## Part E. Concluding thoughts

- What was it that made you agree to take part in this interview study?
- Is there anything that we have not talked about that you would like to raise?
- Thank them for their time, reiterate confidentiality, discuss reimbursement.

## Supplemental material 2

# Explaining randomised controlled trials (RCTs)

## What is an RCT?

- In health care, treatments for a condition (for example, treatment for anxiety in autistic adults) need to be based on evidence from research studies.
- An RCT is a research study that is undertaken when we want to find out if a new or existing treatment works better than another treatment, an inactive treatment (placebo) or no treatment.

## How are treatments allocated in an RCT?

- In an RCT, people are allocated into two or more groups.
- One group will receive the new/existing treatment that is being assessed (called the intervention group) and the other group(s) will receive an alternative treatment, a placebo or no treatment (called the comparison group).
- People are allocated into the intervention or comparison groups using a method called ‘randomisation’.
- Randomisation means that every person has an equal chance of being in the intervention or control group(s).
- We use randomisation because it creates similar groups of people which enables a fair comparison.
- If the person in the study or the doctor chose the treatment, then the groups of people being compared may not be similar. For example, there may be more younger people in the intervention than comparison group. The results may then be biased.

**General population**

**Randomised**

**Intervention group**

**Comparison** **group**

| Randomisation creates similar groups of people to enable a fair comparison of the treatment being assessed. |
| --- |

## What is a placebo?

- In some RCTs, people might be allocated to receive a **‘placebo’**.
- A placebo is an inactive treatment that is designed to look exactly like the real (active) treatment.
- If more people taking the active treatment get better than those taking the placebo, then we know that the active treatment works.

## What is blinding in an RCT?

- In some RCTs, people and researchers will not know which treatment has been allocated. This is called ‘blinding’.
- It is done because if people knew which treatment they were getting it might influence how they felt or how they reported their symptoms.

## Supplemental material 3

Table s1

Demographic information for 49 interviewees.

| ID | Age in years | Gender | Highest educational qualification | Current employment status current | Self-reported autism diagnosis | Year of diagnosis | Interview mode | Duration (minutes) |
| --- | --- | --- | --- | --- | --- | --- | --- | --- |
| 1 | 49 | Female | Postgraduate degree | Part time | Autism Spectrum Disorder | 2018 | Telephone | 60 |
| 2 | 61 | male | Pre-degree | Volunteer | Asperger's Syndrome | 2012 | Online platform | 54 |
| 3 | 27 | Male | Bachelor Degree | Self-Employed | Asperger's Syndrome | 2004 | Online platform | 90 |
| 4 | 33 | female | Postgraduate degree | Unemployed | Asperger's Syndrome | 2015 | Online platform | 40 |
| 5 | 39 | Female | Postgraduate degree | Full time | Asperger's Syndrome | 2015 | Telephone | 70 |
| 6 | 49 | Non Binary | Postgraduate degree | Full time | Asperger's Syndrome | 2016 | Online platform | 40 |
| 7 | 58 | Female | Pre-degree | Unemployed/Disabled | Autism Spectrum Disorder | 2017 | Telephone | 54 |
| 8 | 28 | female | Bachelor Degree | Full time | Autism Spectrum Disorder | 2017 | Email | n/a |
| 9 | 65 | Male | Bachelor Degree | Unemployed | Asperger's Syndrome | 2010 | Telephone | 52 |
| 10 | 33 | Male | Postgraduate degree | Full time | Asperger's Syndrome | 1996 | Telephone | 51 |
| 11 | 51 | Female | Pre-degree | Unemployed | Autism Spectrum Disorder | 2016 | Email | n/a |
| 12 | 34 | Male | Postgraduate degree | Full time | Asperger's Syndrome | 2007 | Email | n/a |
| 13 | 54 | Male | Postgraduate degree | Unemployed | Asperger's Syndrome | 2008 | Online platform | 60 |
| 14 | 46 | Female | Pre-degree | Unemployed | Asperger's Syndrome | 2012 | Telephone | 55 |
| 15 | 21 | Male | Pre-degree | Unemployed | Autism Spectrum Disorder | 2016 | Face-to-face | 54 |
| 16 | 28 | Female | Bachelor Degree | unemployed | Autism Spectrum Disorder | 2018 | Telephone | 42 |
| 17 | 59 | Male | Postgraduate degree | Full time | Asperger's Syndrome | 2008 | Telephone | 60 |
| 18 | 44 | Male | Postgraduate degree | Full time | Autism Spectrum Disorder | 2015 | Telephone | 44 |
| 19 | 58 | Male | Bachelor Degree | Full time | Autism Spectrum Disorder | 2019 | Email | n/a |
| 20 | 37 | Male | Postgraduate degree | Full time | Asperger's Syndrome | 2017 | Telephone | 54 |
| 21 | 54 | Male | Bachelor Degree | Part time | Autism Spectrum Disorder | 2018 | Telephone | 47 |
| 22 | 67 | Female | Bachelor Degree | Retired | Asperger's Syndrome | 2014 | Telephone | 45 |
| 23 | 29 | Male | Postgraduate degree | Student | Asperger's Syndrome | 1996 | Online platform | 46 |
| 24 | 55 | Male | Postgraduate degree | Unemployed/Disabled | Autism Spectrum Disorder | 2020 | Email | n/a |
| 25 | 33 | Male | Postgraduate degree | Full time | Autism Spectrum Disorder | 2018 | Telephone | 28 |
| 26 | 29 | Female | Pre-degree | Full time | Autism Spectrum Disorder | 2018 | Online platform | 35 |
| 27 | 50 | Female | Pre-degree | Part time | Autism Spectrum Disorder | 2016 | Telephone | 55 |
| 28 | 57 | Female | Pre-degree | Unemployed | Autism Spectrum Disorder | 2014 | Telephone | 54 |
| 29 | 39 | Male | Bachelor Degree | Part time | Autism Spectrum Disorder | 2018 | Online platform | 43 |
| 30 | 25 | Male | Bachelor Degree | Full time | Autism Spectrum Disorder | 2016 | Email | n/a |
| 31 | 35 | Male | Pre-degree | Full time | Asperger's Syndrome | 1999 | Email | n/a |
| 32 | 61 | Male | Postgraduate degree | Retired | Asperger's Syndrome | 2017 | Online platform | 65 |
| 33 | 49 | Female | Bachelor Degree | Retired | Autism Spectrum Disorder | 2019 | Telephone | 43 |
| 34 | 49 | Female | Postgraduate degree | Unemployed | Autism Spectrum Disorder | 2009 | Online platform | 23 |
| 35 | 63 | Male | Postgraduate degree | Retired | Asperger's Syndrome | 2008 | Text app | 105 |
| 36 | 27 | Female | Bachelor Degree | Full time | Asperger's Syndrome | 2017 | Online platform | 54 |
| 37 | 36 | Female | Pre-degree | Unemployed | Asperger's Syndrome | 2017 | Online platform | 65 |
| 38 | 63 | Female | Postgraduate degree | Part time | Autism Spectrum Disorder | 2018 | Online platform | 88 |
| 39 | 58 | Female | Postgraduate degree | Part time | Asperger's Syndrome | 2009 | Online platform | 71 |
| 40 | 37 | Female | Bachelor Degree | Student/  Volunteer/parent | Autism Spectrum Disorder | 2018 | Text app | n/a |
| 41 | 26 | Female | Pre-degree | Full time | Autism Spectrum Disorder | 2019 | Online platform | 42 |
| 42 | 52 | Female | Bachelor Degree | Full time | Asperger's Syndrome | 2016 | Telephone | 65 |
| 43 | 67 | Male | Bachelor Degree | Retired | Asperger's Syndrome | 2014 | Telephone | 49 |
| 44 | 44 | Male | Postgraduate degree | Full time | Autism Spectrum Disorder | 2018 | Telephone | 53 |
| 45 | 44 | Female | Pre-degree | Self-Employed | Asperger's Syndrome | 2014 | Email | n/a |
| 46 | 66 | Male | Bachelor Degree | Full time | Asperger's Syndrome | 2010 | Telephone | 79 |
| 47 | 27 | Non-binary | Pre-degree | Unemployed/Disabled | Asperger's Syndrome | 2011 | Online platform | 62 |
| 48 | 28 | Non-binary | Postgraduate degree | Student/  part-time researcher | Autism Spectrum Disorder | 2018 | Online platform | 51 |
| 49 | 31 | Male | Pre-degree | Unemployed | Asperger's Syndrome | 1999 | Telephone | 58 |

## Supplemental material 4

Table s2

Extra illustrative quotes

| Theme | Quote |
| --- | --- |
| Bio psychosocial context | Same as NT populations  *“if you’re talking about autistic adults who are falling within normal intelligence or the cognitive skill ranges, then you might be running a pretty standard trial.” (P4).*  Social interaction and communication issues  *I still have certain challenges, mainly the social aspects, managing relationships and friendships can be quite difficult at times. Also, navigating new and unknown environments, I can easily get lost. That causes frustration and anxiety. Occasional feelings of burnout, if things become too overwhelming then it’s wanting to close down for some time and try to recover from that in private. (P17)*  Sensory issues  *“low pitched electric sounds can just completely send me into meltdown, very quickly” (P47)*  Masking  *“incredible amount of work I’ve had to do to fit in and to change my myself in order to be safe, essentially, and be able to function within the world” (P32)*  Routines  *“routines are helpful because I know what to expect. They're also helpful for remembering a lot of daily chores and things because daily tasks can be overwhelming sometimes”* (P8). |
| Altruism | Moral obligation  *It was my duty to do it [participate in research], regardless of the topic. I feel obliged with a medical condition to try and improve research and help with research. (P33)*  Direct benefit to the autistic community  *I like to try to better understand my mental health and find this sort of thing aids my thinking. (P24)*  *Try and find a way to motivate and inform autistic people that it's in their best interests and while there is no cure for ASC some help with relieving the stress, anxiety and depression that often afflict us would be most welcome. (P31)*  Informing the general public  *When I had a screening phone call, because I’d declared I was on the autistic spectrum, they said that was a screen out factor. That made me ineligible to take part. I think in many cases, for medical research, autistic people won’t be able to participate because criteria state ASC or ASD is a medical condition that won’t allow you to be involved unfortunately. I don’t know if it will change over time (P3).* |
|  | Distrusting medical professionals and medical research.  *I feel very vulnerable (…) The thought of doing anything when I don’t know what the affect might be and I don’t expect the health professional to be able to effectively advise me (…) To be perfectly honest, I try to see the doctor only when I think I have serious health problems. (P5)*  *data fudging reports based on unreliable research on how psychotropic medication interacts with people on the autistic spectrum (P37).*  *What I would want to know what the aim [of the study] was to make people who are autistic feel less anxious, why that was the design of that outcome because if it was a desirable outcome because people who are trying to make people less anxious are actually trying to in some way cure the autism, it doesn’t feel very altruistic (P18)* |
|  | Expecting communication barriers  *Yes [I would find difficult to explain what an RCT is], but for me, that's the case with most things. I was reading 'The Theory of Everything' and trying to explain that. I got it in my head. I understood it, but I couldn't actually get it out. (P7)*  “*I get sensory overload from noise and light in the wrong place, out of place. That can affect me quite badly” (P2)*  *“I would struggle getting to (city) anyway. I don’t like going to (city)”(P13).* |
|  | Introducing uncertainty  *Now this is tricky for me, I like to feel that I am in control of a situation. So being assigned to a group via blinding would be a struggle & not knowing whether or not I was allocated to a particular group! It kind of fails my "control" test, so on balance I probably would not feel comfortable with it. (P24)* |
|  | Blinding & randomisation  Fair & honest processes  *“they don’t know exactly what [they are taking], to make it fair researchers would have to make sure nobody knows who is taking tablets and who isn’t”* (P2).  Being tricked  *I would not participate in such an experiment. Partly because of having, maybe, a thought of it being trickery. If you gave somebody a placebo, by definition it could only have a psychological effect. If you gave somebody a placebo and didn't tell them, then that's trickery. (P9)*  *You have to be able to make the decision for yourself. It can’t be influenced by parents or carers or others in your life. You have to be clear in your intention (…) Autistic people do have circumstances in their lives that are much more challenging and a bit more harsh, compared to the wider population, especially around anxiety or depression, for example. Their mental health needs are somewhat more exacerbated and a bit more challenging. (P3)*  Active treatment  *If you are in a relatively stable position, your anxiety has not got to a point where it’s severely affected your mental health but at the same time you feel that the medication either way, taking it or not, is not going to create an adverse side-effect for your own wellbeing then I might feel a bit more willing to participate* (P3)  Placebo effect  *Because ultimately I haven't taken anything, nothing has changed and if psychologically I thought that I was improving then great, better quality of life for no drugs.(P26)* |
| Co-production | *we should be doing that research because it’s about our community. […] We have such an immense amount of empathy and we have a very much greater instinctive empathy for other autistic people.(P10)* |
| Reducing uncertainty | Mind the language  *I think it’s necessary to be as transparent as possible […] ways in which [research] is funded…(P23)*  *they will use questionnaires designed for neurotypical people on autistic people. They also don't take into account other disabilities that autistic people could have, for example there's always a question about sleep on mental health surveys, and there's nowhere to put that I have a sleep disorder. (P8)*  Detailed and easy to read  *I would definitely want to know that I could have some kind of shortcut, or hotline to accessing professional help quickly related to the trial if I was experiencing any serious side effects. (P4)*  Precise language  *The sentence, ‘We use randomisation because it creates similar groups of people, which enables a fair comparison.’ I think if it’s completely random then you can’t necessarily say you’re going to create similar groups of people, because you just don’t know. I can see where that’s coming from because it’s, I would imagine, trying to make the point (P1)*  *Satisfaction and commitment*  *There's a level of uncertainty involved in - you don't know whether what you're taking is the active substance or the placebo. But you've agreed to do it. (P29)* |
